# Fat Embolization Syndrome Secondary to Steroid Treatment in a Case of Sickle Cell Vaso-Occlusive Crisis

**DOI:** 10.1155/2023/5530870

**Published:** 2023-07-03

**Authors:** Ram Prakash Thirugnanasambandam, Farish Mohamed Maraikayar, Marie Liu, Khalid Elbashir, John Muthu

**Affiliations:** ^1^Department of Internal Medicine, SUNY Downstate Medical Center, Brooklyn, NY 11203, USA; ^2^Medical Student, SUNY Downstate Medical Center, Brooklyn, NY 11203, USA; ^3^Department of Adult Sickle Cell Practice, NYC Health + Hospitals/Kings County, Brooklyn, NY 11203, USA

## Abstract

Fat embolization syndrome (FES) is often seen as a complication of fractures and has been known to cause respiratory failure, rashes of the skin, thrombocytopenia, and neurological damage. Nontraumatic FES is uncommon and occurs due to bone marrow necrosis. Vaso-occlusive crisis in sickle cell patients secondary to steroid therapy is a rare entity and not widely acknowledged. We report a case of FES secondary to steroid therapy administered for a patient with intractable migraine. FES is an uncommon yet serious complication that occurs due to bone marrow necrosis and is usually associated with increased mortality or damaging neurologic sequelae for the surviving patient. Our patient was initially admitted for intractable migraine and worked up to rule out any acute emergency conditions. She was then given steroids for her migraine which did not subside with the initial treatment. Her condition worsened, and she developed respiratory failure along with altered mental status requiring care in the intensive care unit (ICU). Imaging studies showed microhemorrhages throughout the cerebral hemispheres, brainstem, and cerebellum. The imaging of her lungs confirmed severe acute chest syndrome. The patient also had hepatocellular and renal injuries indicative of multiorgan failure. The patient was treated with a red cell exchange transfusion (RBCx) leading to an almost complete recovery in a few days. The patient, however, had residual neurological sequelae with the presence of numb chin syndrome (NCS). This report thus highlights the need to recognize potential multiorgan failure secondary to steroid treatment and the importance of initiating treatment with red cell exchange transfusions to decrease the risk of such complications secondary to steroids.

## 1. Introduction

Vaso-occlusive crises are the commonest cause of hospitalization in patients with sickle cell disease [1]. The use of systemic corticosteroids has been considered a possible risk factor for vaso-occlusive crisis (VOC) and has also been debated as a possible treatment for VOC, including acute chest syndrome [2–4]. In sickle cell disease (SCD), there is a chronic phenomenon due to bone marrow necrosis leading to the release of fat droplets into the circulation which has been proven to be due to the elevated levels of secretory phospholipase A2 (sPLA2) which is seen in a steady state in SCD patients when compared to normal individuals [5]. The characteristic picture of multiorgan failure is due to fat embolization syndrome (FES) which occurs as a result of bone marrow necrosis which leads to the release of large amounts of fat droplets that enter systemic circulation. However, fat embolization syndrome in SCD is rare, with just a few cases reported worldwide [6]. We report a case of FES secondary to steroid therapy administered for an unrelated condition and an almost complete recovery after red cell exchange transfusion (RBCx).

## 2. Case Summary

A 27-year-old woman with sickle cell disease (HbSC genotype) and a history of migraine presented to our hospital with complaints of intractable headaches. She was diagnosed by our neurologist as having status migrainosus, and the neurologist proceeded to rule out other causes of her headache. Approximately 12 days before her current hospitalization, she received a Pfizer COVID-19 vaccine, following which she developed headaches, chills, shortness of breath, and left arm swelling (at the site of her injection). Except for her headache, the other symptoms had subsided by the time she was evaluated. Subarachnoid hemorrhage (SAH) was ruled out by lumbar puncture (LP), although she was noted to have an elevated CSF opening pressure of 31 cm. Magnetic resonance imaging (MRI) of the brain performed on day 2 revealed small focal areas of increased T2 signals in bilateral frontal and parietal subcortical and deep white matter, consistent with migraine. Magnetic resonance angiography (MRA) was negative for intracranial aneurysms or stenosis. Magnetic resonance venography (MRV) was negative for cerebral venous sinus thrombosis. The patient was tried on various treatments, including prednisone (60 mg × 5 days), valproate, amitriptyline, and topiramate unsuccessfully for status migrainosus; acetazolamide for suspected idiopathic intracranial hypertension was administered.

The patient developed acute pain in the neck and shoulders the day following steroid administration, consistent with a sickle vaso-occlusive crisis. The MRI scan of the cervical spine performed subsequently was negative for cervical spondylosis. On day 5, the patient received a sphenopalatine block for her intractable headache, which did not help. Hematology consultation was obtained on day 6 for the sickle vaso-occlusive crisis, which recommended optimizing her pain regimen with ketorolac (dose increased from 15 to 30 mg every 8 hours intravenously), and prednisone was recommended to be discontinued as it was considered a possible causative factor for a sickle cell crisis. Transfusions were not indicated at this time for her uncomplicated pain crisis. On day 7, her pain crisis continued to progress to multiple locations with poor pain control. She was also noted to be tachypneic with a respiratory rate of 30 and subsequently developed type 1 acute hypoxic respiratory failure and was transferred to the intensive care unit (ICU).

Laboratory parameters revealed a drop in hemoglobin from 11.8 g/dL to 6.6 g/dL, and the platelet count dropped from 238 K/uL to 35 K/uL over a period of 4 days. Hemolytic indicators decreased from 84 mg/dl to <10 mg/dl, and the LDH level increased from 172 U/L to >1800 U/L, while bilirubin elevation (from 0.8 mg/dL to 1.4 mg/dL) did not commensurate with the elevation of LDH. Other salient laboratory abnormalities were elevated levels of aspartate transaminase (AST) (from 9 U/L to a peak of 118 U/L), alkaline phosphatase (ALP) (from 64 U/L to a peak of 288 U/L), and ammonia levels which rose to 46 mmol/L. After these laboratory derangements were noted, amitriptyline and valproate were discontinued which correlated with a partial improvement in liver function enzymes (AST decreased to 41 U/L and ALP to 87 U/L). Prothrombin time (PT) was elevated starting on day 10 (at 18.8 seconds) suggesting multiorgan dysfunction. The ammonia elevation to 46 mmol/L was mild and did not explain the patient's altered mentation. Computed tomography (CT) without contrast was performed on suspicion of stroke which showed a subtle small focus of hypoattenuation in the right corona radiata extending into the basal ganglia, not definitively seen on the prior CT or MRI scan performed one week before. MRI of the brain showed interval development of innumerable numbers of small foci of low signal intensities throughout the cerebral hemispheres, brainstem, and cerebellum most consistent with microhemorrhages, and these were not present in prior MRI performed on day two. These findings suggested that the patient's hypoxic respiratory failure and subsequent acute chest syndrome were likely secondary to FES. The patient received an RBCx with 5 units of packed red cells on day 15, and an HbA level of 74% was achieved. Following RBCx, the hemolytic parameters started improving. The patient's mentation improved, she was noted to be alert and oriented to time, place, and person, and she was subsequently discharged home. The patient had residual mental neuropathy in the form of numb chin syndrome on subsequent clinic follow-up.

## 3. Discussion

The first case of FES in a patient with sickle cell disease was reported in 1941 [14]. While FES is often seen as a complication of fractures and has been known to cause respiratory failure, rashes of the skin, thrombocytopenia, and neurological damage [6], nontraumatic FES is uncommon and occurs due to bone marrow necrosis. While FES in the setting of sickle VOC is reported, we wanted to highlight the likelihood of steroids as a potential cause of FES [15]. A thorough literature review was conducted on PubMed using the following keywords: “fat embolism in sickle cell,” “neurological sequelae of fat embolism in sickle cell,” “presentation of fat embolism in a sickle cell patient,” and “treatment of fat embolism in sickle cell.”

Table 1 shows a comprehensive literature review of the common characteristics of FES in sickle cell disease since 2020. There have been seven FES cases reported since 2020. Of those seven cases, four were men and three were women, corresponding to 57% and 43%, and the age range was between 27 and 75, thus indicating that FES can occur at any age among patients with sickle cell disease. Of the seven cases reported in Table 1, 71% were younger than 40 years and 29% were older than 60 years.

The most common presentation of FES secondary to sickle VOC as noted in Table 1 includes fever (43%), joint pain or body aches (86%), and altered mental status (43%) [16]. FES commonly presents as a triad of hypoxemia, neurological abnormalities, and thrombocytopenia and is usually seen within 1–3 days after the initial insult [17]. In our case, the patient developed respiratory failure around day 7, needing ICU-level care. FES is a life-threatening complication seen in the setting of sickle cell VOC, often leading to multiorgan failure with a high rate of mortality and morbidity [18].

CNS manifestations of sickle cell disease include headaches, ischemic and hemorrhagic strokes, intracerebral hemorrhage, subarachnoid hemorrhage (SAH), cerebral venous thrombosis, cerebral aneurysm, and Moyamoya syndrome [19]. Acute stroke as well as other neurological complications in a patient with sickle cell disease needs emergency management to prevent a permanent neurologic deficit. Our patient was seen in the emergency department for presumed migraine, while other differentials were considered, including cerebral aneurysm and cerebral venous thrombosis.

It may often be very difficult to diagnose a patient with sickle cell VOC and concurrent FES, as there may be delays due to similarities seen in sickle cell VOC with multiorgan failure or thrombotic thrombocytopenic purpura occurring concurrently in sickle cell disease [20]. About 57% of the patients in Table 1 had a lumbar puncture as part of their workup. While the diagnosis of FES is clinically based on the presence of the triad of pulmonary symptoms such as hypoxemia, CNS symptoms such as altered mental status, and petechiae, it is important to note that all findings may not be present at the outset and that the diagnosis is usually reached with a high degree of suspicion. A study conducted by Bailey et al. on FES, in general, has concluded that it is useful to use either the Gurd and Wilson or the Schonfeld fat embolism criteria as diagnostic tools when there is a high index of suspicion for FES [21].

**Figure 1:**
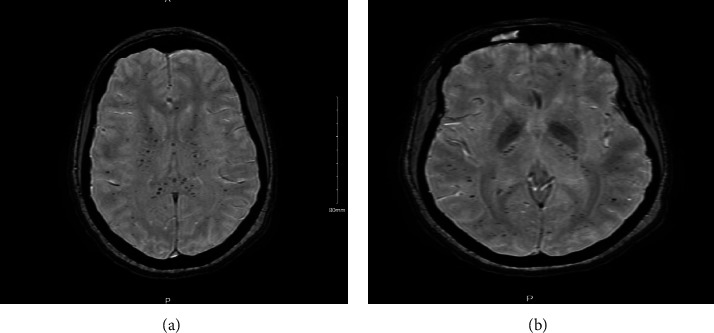
MRI: axial susceptibility weighted sequence showing innumerable small foci of low signal throughout the cerebral hemispheres, brainstem, and cerebellum most consistent with microhemorrhages.

The diagnosis of sickle cell FES is made by a high degree of clinical suspicion along with supportive imaging as mentioned in the following discussion. Imaging tests play a vital role in diagnosis due to the inability to rely on signs and symptoms with complete certainty. The CT scan of the brain may show edema with possible scattered areas of low attenuation and hemorrhage in a few cases. However, in most patients, the CT findings are normal though they may have features of encephalopathy and neurological deficits [22]. Thus, an MRI scan of the brain is primary imaging used to diagnose CNS involvement of FES as seen in all patients in Table 1, and it typically shows a “starfield pattern” which has been reported in every single patient listed including our patient as shown in Figure 1 [23]. It may be noted that the starfield pattern is associated with a good prognosis despite severe neurological insult. A bone marrow biopsy though not absolutely needed to be performed for diagnosis may aid in the diagnosis and is seen to have been performed among 43% of our patients in Table 1. Findings may include extensive necrosis, stromal hemorrhage, microvascular thrombi, and multiple sickle cells in marrow sinuses and stroma [24]. However, it is important to remember that these findings are not often seen, and the timing of the biopsy is important as there is a rapid recovery of the marrow after an acute injury.

The mainstay of treatment of FES among most cases seen in the literature includes supportive care and exchange transfusion. RBCx when initiated early with the suspicion of FES helps prevent mortality among patients [25]. In Table 1, 100% of all patients received RBCx as part of their treatment, thus underlining the importance of the intervention. There has been support for the use of plasma exchange in recent years with the rationale being that it may help by removing fat droplets and cytokines released from bone marrow necrosis [26, 27].

Our patient had an immediate recovery with RBCx along with supportive care at the intensive care unit. Most patients recover completely with RBCx and supportive treatment. Mortality from FES is typically 5–15%, with respiratory failure being a leading cause of death [28]. While patients may experience neurological symptoms of FES, these are reversible over time as seen in the literature [29, 30]. In our analysis of similar case reports of FES in the literature, as shown in Table 1, one patient experienced psychomotor retardation, while another had quadriparesis. All other patients recovered with no complications. Our patient suffered numb chin syndrome secondary to mental neuropathy, which has continued to gradually improve since discharge.

## 4. Conclusion

This case highlights the need to have a high index of clinical suspicion to diagnose sickle cell VOC complicated by FES promptly in the presence of a typical constellation of clinical findings. FES is a devastating complication of sickle cell disease resulting from extensive bone marrow necrosis and is associated with high mortality rates, while survivors may suffer from severe neurological sequelae. FES in the sickle cell remains underrecognized. It is also important to highlight the risk of FES in sickle cell secondary to corticosteroid therapy. More studies are needed to evaluate the safety of corticosteroid use in patients with sickle cell disease in the light of several case reports [2, 15, 31–33].

## Figures and Tables

**Table 1:** The following keywords were used for a literature search in PubMed: “fat embolism in sickle cell,” “neurological sequelae of fat embolism in sickle cell,” “presentation of fat embolism in sickle cell patients,” and “treatment of fat embolism in sickle cell.”

Article titles	Age (in years) and sex of the patient	Initial symptoms	Presentation on imaging	Procedure	Treatment and complications	Ref. no
Fat embolism in sickle-cell disease: A case report with literature review	37 females	Lower back pain and bilateral shoulder pain	CT/CTA reports were normal	Lumbar puncture	Red cell exchange transfusion	[7]
PMID: 36741476			Brain MRI showed a “starfield appearance” and incidental aneurysm in the supraclinoid portion of the right ICA		Psychomotor slowness in long-term follow-up	

Bone marrow necrosis and fat embolism syndrome in sickle cell disease during COVID-19 infection treated successfully with sequential red cell and plasma exchange	35 males	Generalized body aches, altered mental status, and fever	Brain MRI showed multiple areas of restricted diffusion and microhemorrhages in a so‐called “starfield pattern”	Lumbar puncture and bone marrow biopsy	Red cell exchange transfusion and plasma cell exchange transfusion	[8]
PMID: 36718354					No complications	

A suspected case of cerebral fat embolism triggering a drug-resistant status epilepticus in a HbS/*β*+-thalassaemia patient	61 females	Severe precordial chest pain and dyspnea	Brain MRI showed widespread, nonconfluent areas of ischemia in multiple anterior and posterior vascular distributions with microhemorrhages consistent with a “starfield appearance” with a “walnut kernel microbleed” pattern	Lumbar puncture	Red cell exchange transfusion therapy	[9]
PMID: 35444771					No complications	

Complete neurological recovery from fat embolism syndrome in sickle cell disease after sequential red cell exchange transfusion and therapeutic plasma exchange	27 males	Altered mental status	Brain MRI showed multiple widespread microhemorrhages showing not only the characteristic “starfield” pattern but also a cytotoxic lesion of the corpus callosum, known to be the result of direct neurotoxicity by proinflammatory cytokines	Nil	Red cell exchange transfusion	[10]
PMID: 34489185					No complications	

Bone marrow necrosis and fat embolism syndrome: a near-fatal complication in previously undiagnosed sickle beta + thalassemia	37 males	Fever, altered mental status, low back pain, unsteady gait, and urinary incontinence	Brain MRI showed evidence of subacute ischemia and chronic microhemorrhages	Lumbar puncture and bone marrow biopsy	Red cell exchange transfusion	[11]
PMID: 33408108			CT scan of the abdomen and pelvis showed moderate splenomegaly, hepatomegaly, cholelithiasis, and diffuse patchy sclerotic osseous abnormalities		No complications	

Fat embolism syndrome in sickle cell *β*-thalassemia patient with osteonecrosis: an uncommon presentation in a young adult	34 males	Fever, hypoxia, encephalopathy, and generalized body aches	Brain MRI with and without contrast revealed extensive punctate multifocal areas of diffusion restriction throughout the basal ganglia, thalami, and white matter of both hemispheres consistent with multifocal areas of punctate ischemic infarction in the acute to subacute stages	BM biopsy	Red cell exchange transfusion	[12]
PMID: 34008428				Quinton catheter was placed for lifelong exchange transfusions every 4 to 6 weeks to keep hemoglobin S level to a goal of <30%	No complications	

Neurologic recovery in systemic nontraumatic fat embolism syndrome in an elderly patient with hemoglobin SC disease: a case report	75 females	Chest pain and diffuse body aches	Brain MRI showed extensive multiple microhemorrhages across the neuroparenchyma in a “starfield” pattern classic for cerebral FES	Nil	Red cell exchange transfusion	[13]
PMID: 32983503					She remained profoundly paretic and required ventilation and assisted feeding via tracheostomy and gastrostomy	

## Data Availability

The data used to support the findings of the study are included within the article.
